# The Forgotten Hemodynamic (PCO2 Gap) in Severe Sepsis

**DOI:** 10.1155/2020/9281623

**Published:** 2020-01-07

**Authors:** Zouheir Ibrahim Bitar, Ossama Sajeh Maadarani, AlAsmar Mohammed El-Shably, Ragab Desouky Elshabasy, Tamer Mohamed Zaalouk

**Affiliations:** Critical Care Unit, Ahmadi Hospital, Kuwait Oil Company, P.O. Box 46468, Postal Code 64015, Ahmadi, Kuwait

## Abstract

**Background:**

Central venous-arterial carbon dioxide difference (PCO2 gap) can be a marker of cardiac output adequacy in global metabolic conditions that are less affected by the impairment of oxygen extraction capacity. We investigated the relation between the PCO2 gap, serum lactate, and cardiac index (CI) and prognostic value on admission in relation to fluid administration in the early phases of resuscitation in sepsis. We also investigated the chest ultrasound pattern A or B.

**Method:**

We performed a prospective observational study and recruited 28 patients with severe sepsis and septic shock in a mixed ICU. We determined central venous PO2, PCO2, PCO2 gap, lactate, and CI at 0 and 6 hours after critical care unit (CCU) admission. The population was divided into two groups based on the PCO2 gap (cutoff value 0.8 kPa).

**Results:**

The CI was significantly lower in the high PCO2 gap group (*P*=0.001). The high PCO2 gap group, on admission, required more administered fluid and vasopressors (*P*=0.01 and *P*=0.009, respectively). There was also a significant difference between the two groups for low mean pressure (*P*=0.01), central venous O2 (*P*=0.01), and lactate level (*P*=0.003). The mean arterial pressure was lower in the high PCO2 gap group, and the lactate level was higher, indicating global hypoperfusion. The hospital mortality rate for all patients was 24.5% (7/28). The in-hospital mortality rate was 20% (2/12) for the low gap group and 30% (5/16) for the high gap group; the odds ratio was 1.6 (95% CI 0.5–5.5; *P*=0.53). Patients with a persistent or rising PCO2 gap larger than 0.8 kPa at *T* = 6 and 12 hours had a higher mortality change (*n* = 6; in-hospital mortality was 21.4%) than patients with a PCO2 gap of less than 0.8 kPa at *T* = 6 (*n* = 1; in-hospital mortality was 3%); this odds ratio was 5.3 (95% CI 0.9–30.7; *P*=0.08). The PCO2 gap had no relation with the chest ultrasound pattern.

**Conclusion:**

The PCO2 gap is an important hemodynamic variable in the management of sepsis-induced circulatory failure. The PCO2 gap can be a marker of the adequacy of the cardiac output status in severe sepsis. A high PCO2 gap value (>0.8 kPa) can identify situations in which increasing CO can be attempted with fluid resuscitation in severe sepsis. The PCO2 gap carries an important prognostic value in severe sepsis.

## 1. Introduction

Severe sepsis remains associated with high mortality, and the early recognition of the signs of tissue hypoperfusion is crucial in its management [1]. The effectiveness of oxygen-derived parameters as resuscitation goals has been questioned, and the latest data have failed to demonstrate clinical advantage [2]. The venous oxygen saturation (SvO2) is <70% in the majority of patients with severe sepsis on admission to the intensive care unit (ICU) [3]. The venous-to-arterial carbon dioxide difference (Pv-aCO2) can indicate the adequacy of microvascular blood flow in the early phases of resuscitation in sepsis [4]. Hence, other resuscitation goals, such as PCO2 gap, have been suggested, due to their ability to predict adverse clinical outcomes and simplicity in patients achieving normal oxygen-derived parameters during the early phases of resuscitation in septic shock.

The optimization of cardiac output during the early resuscitation of patients with sepsis is an important goal in the management of severe sepsis. The Pv-aCO2 usually does not go beyond 0.8 kPa (6 mmHg) under physiological conditions [5] and indicates the adequacy of venous blood flow and cardiac output (CO) [6]. At the circulatory level, an inverse relationship between the PCO2 gap and cardiac index (CI) has been described in critically ill patients with severe sepsis [7]. Combining ScvO2 values as a surrogate for global tissue hypoxia and the PCO2 gap as a substitute for CI may be practical during resuscitation of critically ill patients. However, in patients with sepsis, microcirculatory level distributive changes may be independent of CI [8]. This means that, on a regional level, in agreement with the persistent tissue hypoxia despite normal ScvO2 levels, the accumulation of carbon dioxide (CO2) may occur in sepsis despite adequate CI.

We studied the relationship between the Pv-aCO2and CI and tackled the question of whether the Pv-aCO2 can add a value to the outcome prediction.

### 1.1. Study Design

This work was a prospective, blinded, observational study of an enrolled sample of patients with sepsis who required ICU admission.

### 1.2. Population and Setting

The study was performed in the Ahmadi Hospital critical care unit (CCU). The hospital is a subsidiary of the Kuwait Oil Company (KOC). The hospital serves KOC members and their families, including their parents, with an annual ED census of 60,000 visits. Ultrasound examinations are currently used in daily practice in the CCU. The protocol was reviewed and approved by an Ahmadi Hospital Ethical Committee.

Patients were included if they were >18 years old and had sepsis according to international guidelines [9] between September 2018 and March 30, 2019. Additionally, only patients for whom there was a clinical indication for additional hemodynamic monitoring using transthoracic echocardiographic cardiac output [10] and ultrasound of the chest [9] were included. Informed consent was obtained from the patient or legal representative. Central lines and arterial lines were inserted into every patient admitted to the CCU. Cardiac output and index were measured by a certified intensivist in critical care ultrasound who was blinded to the blood results. Hemodynamic data were collected at the time of admission to the CCU and then every 6 hours (*T*0, *T*1, and *T*2).

Cardiac output was measured by the assessment of stroke volume (SV) by PW Doppler. The site for measurement was the left ventricular outflow tract (LVOT) [10], where SV is the multiplication of the cross-sectional area (CSA) of the LVOT by the velocity-time integral (VTI) at that same location (SV ¼ CSA × VTI). VTI is the sum of the instantaneous velocities measured at a specific location by PW Doppler and is equal to the area under the curve of the Doppler velocity profile. It can be thought of as the distance the average blood cell travels during systole [10]. To calculate CO, the following steps are required [10]:

1. Measure the diameter (*D*) of the LVOT. Obtained from the image of the surface parasternal long-axis view, or the transesophageal route is used when the parasternal view is not clear. Start the measurement from the junction of the atrioventricular leaflet with the septal endocardium to the junction of the other leaflet with the mitral valve posteriorly, using inner edge to inner edge, in early systole [7]. Use the largest of 3 to 5 repeated measurements to avoid underestimation of the true diameter.
2. Calculate the CSA by using the following formula: CSA = *D*2 × 0.785.
3. Measure the velocity at the LVOT using an apical 3-chamber or 5-chamber view. Put the PW sample volume 5 mm above the aortic valve and record a Doppler waveform with a well-defined envelope.
4. Take the average of 3 VTI measurements by tracing 3 consecutive waveforms.
5. Calculate the SV using the following formula: SV = CSA × VTI, and then multiply the SV with heart rate to calculate the CO.

Radiometer ABL90 Flex analyzer (Copenhagen, Denmark) is used to analyze blood samples in the ICU, to ensure rapid assessment and avoid poor quality measurements. The severity score Simplified Acute Physiologic Score (SAPS) was calculated.

### 1.3. Statistics

The admitted patients were divided into two groups: patients with a normal PCO2 gap (<0.8 kPa) and patients with a high PCO2 gap (>0.8 kPa) at *T*0. The two-tailed statistical tests were performed by the Statistical Package for the Social Sciences (IBM SPSS 19). Student's *t*-test was used to assess the differences between both groups in the case of a normal distribution. The chi-square test or Fisher's exact test was used for categorical data. For each time point (*T*0–*T*4), the difference between arterial CO2 partial pressure (paCO2) and central venous CO2 partial pressure (pvCO2) (i.e., the PCO2 gap) was calculated. Additionally, for each time point, the average CI, mean arterial pressure (MAP), ScvO2 lactate, and infusion rate of norepinephrine were calculated. The agreement between the CI and the PCO2 gap was assessed by the mean bias and 95% limits of agreement (mean bias ± 1.969 standard deviation). The odds ratio for mortality between patients with a high PCO2 gap and patients with a low PCO2 gap at both *T*0 and *T*4 was calculated. Data are displayed as the mean ± SD. Statistical significance was assumed at *P* < 0.05.

## 2. Results

We enrolled 31 patients. Of these, three patients were excluded due to lack of consent. Baseline characteristics and outcome of the total population and both groups are shown in Table 1.

**Figure 1:**
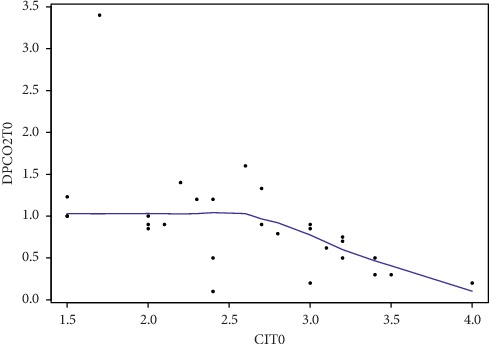
Correlation between central venous PCO2 difference and cardiac index in the total population for all paired measurements at *T* = 0. DPCO2T0: deltaPCO2 on admission; CIT0: cardiac index on admission.

When the CI was plotted against the PCO2 gap for all paired measurements (Figure 1), there was an inverse logarithmic relationship with increasing central PCO2 gap as CI decreased (*R*2 = 0.07; *P*=0.001). CI was significantly lower in the population with a high PCO2 gap (*P*=0.001).

The high CO2 gap group, on admission, required more bolus fluid administration and vasopressors (*P*=0.01 and *P*=0.009, respectively) (Table 1). There was also a significant difference between the two groups for low mean pressure (*P*=0.01), central venous O2 (*P*=0.01), and lactate level (*P*=0.003). The mean arterial pressure was lower in the high CO2 gap group, and the lactate level was higher, indicating global hypoperfusion.

The hospital mortality rate for all patients was 24.5% (7/28). The in-hospital mortality rate was 20% (2/12) for the low gap group and 30% (5/16) for the high gap group; the odds ratio was 1.6 (95% CI 0.5–5.5; *P*=0.53). Patients with a persistent or rising PCO2 gap larger than 0.8 kPa at *T* = 6 and 12 had a higher mortality change (*n* = 6; in-hospital mortality was 21.4%) compared to patients with a PCO2 gap less than 0.8 kPa at *T* = 6 (*n* = 1; in-hospital mortality was 3%); this odds ratio was 5.3 (95% CI 0.9–30.7; *P*=0.08).

The PCO2 gap had no relation with the chest ultrasound pattern.

## 3. Discussion

The venous and arterial PCO2 are dependent on circulatory flow and pulmonary gas exchange, respectively [11]. Thus, a decreased flow will increase the difference between the arterial and venous PCO2 [12]. The cardiac output is measured from the ratio between alveolar oxygen uptake and the oxygen content difference between arterial-venous blood (cardiac output = VO2/CaO2CvO2) or from the ratio of carbon dioxide production and arteriovenous carbon dioxide content difference (cardiac output = VCO2/CaCO2CvCO2), when applying the Fick principle to cardiac output. By substituting CO2 pressure for content, the inverse relationship between cardiac output and the PCO2 gap can be seen [13]. An increase in the venous-arterial PCO2 difference occurs in states of decreased flow, regardless of the reason for the circulatory failure, and this has been demonstrated in clinical trials to be inversely related to cardiac output [14].

In critically ill patients of a mixed population, Cuschieri et al. [7] found a significant inverse relation between the PCO2 gap and CI, taking into consideration that one-third of their population were patients with cardiogenic shock. Our findings are in line with physiological theory, which describes an inverse curvilinear relation between cardiac output and PCO2 gap, according to a modified Fick equation for a range of CO2 production isopleths [15]. Various studies have described such an inverse relationship between the delta PCO2 gap and CI in septic circulatory failure [12, 16]. Due to the heterogeneity of microcirculatory blood flow, inadequate washout of CO2 in microcirculatory weak units, despite normal or even elevated cardiac output, has been observed during sepsis [8]. Vallee et al. [17] tested this hypothesis in patients with septic shock who were supposedly adequately resuscitated at the systemic level, with a ScvO2 C70% [18]. An increase in the PCO2 gap may be a marker of microcirculatory dysfunction in septic shock, especially when SvO2 is normal [4].

The prognostic value of a high PCO2 gap is a matter of debate. There is no mortality difference between the two groups on admission. However, higher mortality is noted in the group with a persistently high PCO2 gap. A persistently elevated PCO2 gap could indicate poor prognosis and nonresponse to current therapy or could indicate severe microcirculatory dysfunction. However, the study was not powered to test whether closing the PCO2 gap will improve the prognosis of the high CO2 gap group.

The chest ultrasound is used for the hemodynamic assessment of shock patients of different etiologies with the need for fluid assessment [19]. This finding depends on the ability of ultrasound to detect interstitial syndrome, which is considered as a direct marker of clinical volume [18]. Our results did not show a significant relation between the chest ultrasound pattern and delta PCO2, taking into consideration that our population is only those with septic circulatory failure. Most likely, delta PCO2 reflects microcirculatory dysfunction, and the chest ultrasound pattern reflects intracardiac pressure.

The small number of patients and the fact that this work is a single-center study are both limitations of the study, but we tried to be blinded to avoid bias. Additionally, the population was only those with severe sepsis or septic shock, and the results cannot be applied to other less critically ill patients or those with shock due to other etiologies.

## 4. Conclusion

The PCO2 gap is an important hemodynamic variable in the management of sepsis-induced circulatory failure. The PCO2 gap can be a marker of the adequacy of cardiac output in severe sepsis. A high PCO2 gap value (>0.8 kPa) can identify situations in which increasing CO can be attempted with fluid resuscitation in severe sepsis. The PCO2 gap carries an important prognostic value in severe sepsis.

## Figures and Tables

**Table 1:** Baseline characteristics.

	Total population (*n* = 28)	Low gap (*n* = 12)	High gap (*n* = 16)	*P* value
Age (years)	71 ± 14	69 ± 17	73.62 ± 13	0.7
Gender (m/f)		7/5	10/6	
SOFA	10.39 ± 3.9	8.5 ± 3.4	11.75 ± 3.8	0.03^*∗*^
Diagnosis				
Abdominal	8	2	6	
Respiratory	11	6	5	
UTI	6	3	3	
Endo	3	1	2	
Lab and therapy				
Lactate (mmol/L)	2.7 ± 2.3	1.3 ± 0.9	3.76 ± 2.6	0.003^*∗*^
CI (L/min/m^2^)	2.6 ± 0.41	3.2 ± 0.37	2.1 ± 0.46	0.001^*∗*^
Ultrasound chest A/B profiles		8/4	8/8	0.8
Mechanical ventilation	8	5	3	0.38
Renal replacement therapy	5	2	3	0.6
Vasopressor	18	5	13	0.009^*∗*^
Fluid boluses	28	10	18	0.01^*∗*^
Mean BP	60 ± 10	65 ± 9	55 ± 11	0.01^*∗*^
SvO2 (%)	71.9 ± 10.7	73.2 ± 9.1	70.3 ± 6.6	0.01^*∗*^
Hematocrit (%)	31 ± 1	30 ± 5	31 ± 6	0.49

^*∗*^Statistically significant difference. SOFA score: sequential organ failure assessment score; Endo: endocarditis.

## Data Availability

The data used to support the findings of this study are available from the corresponding author upon request.
